# Novel sorafenib analogues induce apoptosis through SHP-1 dependent STAT3 inactivation in human breast cancer cells

**DOI:** 10.1186/bcr3457

**Published:** 2013-08-12

**Authors:** Chun-Yu Liu, Ling-Ming Tseng, Jung-Chen Su, Kung-Chi Chang, Pei-Yi Chu, Wei-Tien Tai, Chung-Wai Shiau, Kuen-Feng Chen

**Affiliations:** 1 Institute of Biopharmaceutical Sciences, National Yang-Ming University, No. 155 Sec. 2, Li-Nong Street, Taipei 112, Taiwan; 2 School of Medicine, National Yang-Ming University, No. 155, Sec. 2, Li-Nong Street, Taipei 112, Taiwan; 3 Division of Hematology and Oncology, Taipei Veterans General Hospital, No. 201, Sec. 2, Shih-Pai Road, Taipei 112, Taiwan; 4 Department of Medicine, Taipei Veterans General Hospital, No. 201, Sec. 2, Shih-Pai Road, Taipei 112, Taiwan; 5 Department of Surgery, Taipei Veterans General Hospital, No. 201, Sec. 2, Shih-Pai Road, Taipei 112, Taiwan; 6 Department of Pathology, St. Martin De Porres Hospital, No. 565, Sec. 2, Daya Road, Chiayi 600, Taiwan; 7 Department of Medical Research, National Taiwan University Hospital, No. 7, Chung-Shan South Road, Taipei 100, Taiwan; 8 National Center of Excellence for Clinical Trial and Research, National Taiwan University Hospital, No. 7, Chung-Shan South Road, Taipei 100, Taiwan

## Abstract

**Introduction:**

Signal transducers and activators of transcription 3 (STAT3) signaling is constitutively activated in various cancers including breast cancer and has emerged as a novel potential anti-cancer target. STAT3 has been demonstrated to be a target of sorafenib, and a protein tyrosine phosphatase Src homology 2-domain containing tyrosine phosphatase 1 (SHP-1) has been demonstrated to downregulate p-STAT3 via its phosphatase activity. Here, we tested the efficacy of two sorafenib analogues, SC-1 and SC-43, in breast cancer cells and examined the drug mechanism.

**Methods:**

Breast cancer cell lines were used for *in vitro* studies. Cell viability was examined by the 3-(4,5-dimethylthiazol-2-yl)-2,5-diphenyltetrazolium bromide (MTT) assay. Apoptosis was examined by flow cytometry and western blot. Signal transduction pathways in cells were assessed by western blot. *In vivo* efficacy of sorafenib, SC-1 and SC-43 was tested in xenografted nude mice.

**Results:**

SC-1 and SC-43 induced more potent apoptosis than sorafenib, in association with downregulation of p-STAT3 and its downstream proteins cyclin D1 and survivin in a dose-dependent manner in breast cancer cell lines (HCC-1937, MDA-MB-468, MDA-MB-231, MDA-MB-453, SK-BR3, MCF-7). Overexpression of STAT3 in MDA-MB-468 cells protected the cells from apoptosis induced by sorafenib, SC-1 and SC-43. Moreover, SC-1 and SC-43 upregulated SHP-1 activity to a greater extent than sorafenib as measured by *in vitro* phosphatase assays. Knockdown of SHP-1 by siRNA reduced apoptosis induced by SC-1 and SC-43. Importantly, SC-1 and SC-43 showed more efficacious antitumor activity and p-STAT3 downregulation than sorafenib in MDA-MB-468 xenograft tumors.

**Conclusions:**

Novel sorafenib analogues SC-1 and SC-43 induce apoptosis through SHP-1 dependent STAT3 inactivation and demonstrate greater potency than sorafenib in human breast cancer cells.

## Introduction

Despite the many chemotherapeutic agents available for treatment, metastatic breast cancer remains a major threat to women’s health worldwide as most tumors eventually become chemotherapy-resistant [1]. The five-year relative survival of stage IV breast cancer has been reported to be 23% in the United States [2]. In recent years, several molecularly targeted agents have become available that have advanced anti-cancer therapy. In particular, the improved outcomes reported for trastuzumab, a monoclonal antibody against the human epidermal growth factor receptor 2 (HER2) in the treatment of HER2-positive breast cancer have highlighted the importance of molecularly targeted therapy development in breast cancers [3].

Signal transducer and activator of transcription 3 (STAT3) is essential for normal breast development and involution and may play an important role in breast carcinogenesis [4]. STAT3 is constitutively activated in many common human cancers, including breast cancers [5,6]. Constitutively activated STAT3 can directly contribute to tumorigenesis, invasion and metastasis, and it has been shown that elevated tyrosine-phosphorylated STAT3 (p-STAT3) correlates with incomplete response to neoadjuvant chemotherapy in stage II breast cancers [5,6]. Activated STAT3 signaling also has been shown to induce expression of survivin expression, a direct downstream target of STAT3 and confer resistance to apoptosis in human breast cancer cells [7]. Moreover, IL-6/STAT3 signaling is required for growth of CD44+CD24- stem cell-like breast cancer cells [8], a type of cells that play an important role in the clinical behavior of triple-negative breast cancer (TNBC) [9]. Collectively, these findings suggest that targeting STAT3 may be a promising anti-cancer strategy.

Interestingly, several protein tyrosine phosphatases that can deactivate STAT3 signaling through direct dephosphorylation of p-STAT3 (Tyr 705) might be useful targets for induction of cancer cell death. These phosphatases include members of the Src homology 2 (SH2)-domain containing the tyrosine phosphatase family (SHP-1 and SHP-2) and protein tyrosine phosphatase 1B (PTP-1B) [10-12]. For example, loss of SHP-1 enhances JAK3/STAT3 signaling in ALK-positive anaplastic large-cell lymphoma and in cutaneous T cell lymphoma [13,14]. Moreover, agents such as betulinic acid [15], boswellic acid [16], gambogic acid [17], dihydroxypentamethoxyflavone [18], butein [19], icariside II (a flavonoid icariin derivative) [20] and 5-hydroxy-2-methyl-1,4-naphthoquinone (a vitamin K3 analogue) [21] that can enhance the SHP-1 pathway (either by induction of SHP-1 expression or by increase of SHP-1 activity) have all shown anti-cancer potential. Recently, we reported that sorafenib sensitizes HCC cells to tumor necrosis factor (TNF)-related apoptosis inducing ligand (TRAIL) through the inhibition of p-STAT3 [22]. We further discovered that sorafenib inhibits p-STAT3 through upregulation of SHP-1 activity and induction of apoptosis in HCC cells [12]. Importantly, we further generated a series of sorafenib analogues that are devoid of raf-1 kinase inhibition [23,24], including several with promising anti-cancer potential due to their demonstrated p-STAT3 inhibition. In particular, SC-1, the first proof-of-principle sorafenib derivative that was engineered through replacement of N-methylpicolinamide by a phenylcyano group, showed abolished effects on raf-1 kinase activity while retaining p-STAT3 repressive activity [24]. Our previous results suggest that SHP-1-dependent STAT3 inhibition is a target of sorafenib and that the activated function of SHP-1 phosphatase that targets STAT3 may be a promising candidate for targeted cancer therapy and drug discovery [12,23,24].

In this study, we report the apoptotic effect and mechanism of two novel sorafenib analogues, SC-1 and SC-43 in breast cancer cells. Sorafenib, SC-1 and SC-43 induced apoptosis in association with downregulation of p-STAT3 and its downstream proteins cyclin D1 and survivin in a dose-dependent manner in breast cancer cell lines (HCC-1937, MDA-MB-468, MDA-MB-231, MDA-MB-453, SK-BR3, MCF-7). The apoptotic effects induced by SC-1 and SC-43 were more potent than those seen with sorafenib. Overexpression of STAT3 in MDA-MB-468 cells protected cells from apoptosis induced by sorafenib, SC-1 and SC-43. Moreover, SC-1 and SC-43 upregulated SHP-1 activity to a greater extent than sorafenib as measured by *in vitro* phosphatase assays. Knockdown of SHP-1 by siRNA reduced apoptosis induced by SC-1 and SC-43. Importantly, SC-1 and SC-43 showed *in vivo* efficacy in MDA-MB-468 xenograft tumors. Therefore, these novel sorafenib analogues, SC-1 and SC-43, induced apoptosis through SHP-1-dependent STAT3 inactivation and demonstrated more potent apoptotic effects than sorafenib in human breast cancer cells.

## Methods

### Reagents and antibodies

Sorafenib (Nexavar) was kindly provided by Bayer Pharmaceuticals (West Haven, CT, USA). Sorafenib analogues SC-1 and SC-43 were synthesized, and their quality was evaluated as described in previous studies [23,24]. Sodium vanadate and specific SHP-1 inhibitor were purchased from Cayman Chemical (Ann Arbor, MI, USA). Antibodies for immunoblotting, such as cylcin D1, p-JAK2, JAK1, JAK2 and poly ADP-ribose polymerase (PARP) were purchased from Santa Cruz Biotechnology (San Diego, CA, USA). SHP-1 and phosphospecific anti-SHP-1 (Tyr536 and Ser591) were purchased from Abcam (Cambridge, MA, USA). Other antibodies such as anti-Mcl-1, survivin, p-STAT3 (Tyr705), STAT3, p-JAK1 and caspase-3 were from Cell Signaling (Danvers, MA, USA).

### Cell culture

The HCC-1937, MDA-MB-231, MDA-MB-468, MDA-MB-453, SK-BR3 and MCF-7 cell lines were obtained from the American Type Culture Collection (Manassas, VA, USA). All breast cancer cells were maintained in (D)MEM medium supplemented with 10% fetal bovine serum, 0.1 mM nonessential amino acids, 2 mM L-glutamine, 100 units/mL penicillin G, 100 μg/mL streptomycin sulfate and 25 μg/mL amphotericin B in a humidified incubator and an atmosphere of 5% CO_2_ at 37°C in air. Lysates of breast cancer cells were treated with drugs at the indicated concentrations for various periods of time.

### Cell viability and proliferation of breast cancer cell lines *in vitro*

Cell viability and proliferation of breast cancer cells treated with or without sorafenib, SC-1 or SC-43 were assessed by colorimetric assay using 3-(4,5-dimethylthiazol-2-yl)-2,5-diphenyltetrazolium bromide (MTT). Cells were plated in a 96-well plate in 100 μl (D)MEM per well and cultured for up to 24 hours. Cells were incubated for four hours at 37°C with MTT; after incubation, medium was removed and cells were treated with dimethyl sulfoxide (DMSO) for five minutes. Viability was evaluated by ultraviolet absorption spectrum at 550 nm with a Microplate Reader Model 550 (Bio-Rad, Richmond, CA, USA).

### Apoptosis analysis

Drug-induced apoptotic cell death was assessed by western blot analysis of caspase 3 activation or PARP cleavage and measurement of apoptotic cells by flow cytometry (sub-G1 analysis).

### Gene knockdown using siRNA

Smart-pool siRNA, including control (D-001810-10), SHP-1, were all purchased from Dharmacon (Chicago, IL, USA). Briefly, cells were transfected with siRNA (final concentration, 100 nM) in six-well plates using lipid-mediated transfection with Lipofectamine2000 (Invitrogen, Life Technologies, Carlsbad, CA, USA) according to the manufacturer’s instructions. After 48 hours, the medium was replaced and the breast cancer cells were incubated with SC-1 or SC-43, harvested and separated for western blot analysis and apoptosis analysis by flow cytometry as described previously [12].

### MDA-MB-468 with ectopic expression of STAT3

STAT3 cDNA was purchased from Addgene plasmid repository (Cambridge, MA, USA; http://www.addgene.org/) and constructed into a pCMV6 vector. MDA-MB-468 cells with ectopic expression of STAT3 derived from a single stable clone were prepared for *in vitro* assay for STAT3 target validation and for *in vivo* xenograft tumor growth. Briefly, following transfection, cells were incubated in the presence of Geneticin (Invitrogen, Life technologies; G418, 0.78 mg/mL). After eight weeks of selection, surviving colonies, that is, those arising from stably transfected cells, were selected and individually amplified.

### Phosphatase and kinase activity assays

The RediPlate 96 EnzChek Tyrosine Phosphatase Assay Kit (R-22067) was used for SHP-1 activity assay (Molecular Probes, Carlsbad, CA, USA). Briefly, breast cancer cell protein extracts were incubated with anti-SHP-1 antibody in immunoprecipitation buffer overnight. Protein G-Sepharose 4 Fast Flow (GE Healthcare, Piscataway, NJ, USA) was added to each sample followed by incubation for three hours at 4°C with rotation and then assayed for phosphatase activity. Raf-1 Kinase Cascade Assay Kit (Upstate-Millipore, Billerica, MA, USA) was used to examine the Raf-1 kinase activity. Briefly, Raf-1 immunoprecipitated from breast cancer cell extracts was incubated with mitogen-activated protein kinase kinase (MEK) recombinant protein and the p-MEK was assayed in the drug-treated cells.

### Xenograft tumor growth

Female NCr athymic nude mice (four to six weeks of age) were obtained from the National Laboratory Animal Center (Taipei, Taiwan, ROC). The mice were housed in groups and maintained in a specific pathogen free (SPF)-environment. All experimental procedures using these mice were performed in accordance with protocols approved by the Institutional Animal Care and Use Committee of Taipei Veterans General Hospital. Each mouse was inoculated subcutaneously in the dorsal flank with 2 × 10^6^ breast cancer cells suspended in 0.1 mL serum-free medium containing 50% Matrigel (BD Biosciences, Bedford, MA, USA) under isoflurane anesthesia. Tumors were measured using calipers and their volumes calculated using a standard formula: width^2^ × length × 0.52. When tumors reached 100 to 200 mm^3^, mice received sorafenib tosylate (10 mg/kg) per os once daily or SC-1 (10 mg/kg) per os once daily or SC-43 (10 mg/kg) per os once daily. Controls received vehicle. On termination of treatment (after 28 days), mice were sacrificed and xenografted tumors were harvest and assayed for molecular events by Western blot analysis and for SHP-1 activity.

For STAT3-overexpressed MDA-MB-468 xenograft tumor growth, cells with ectopic expression of STAT3 derived from a single stable clone were prepared and xenografted in nude mice using the same method for wild-type cells.

### Immunohistochemical staining

Paraffin-embedded breast cancer tissue sections (4-μm) on poly-1-lysine-coated slides were deparaffinized and rinsed with 10 mM Tris–HCl (pH 7.4) and 150 mM sodium chloride. Peroxidase was quenched with methanol and 3% hydrogen peroxide. Slides were then placed in 10 mM citrate buffer (pH 6.0) at 100°C for 20 minutes in a pressurized heating chamber. After incubation with 1:50 dilution of anti-SHP-1 antibody (rabbit monoclonal to SHP-1 (ab32559), Abcam) and with 1:50 dilution of anti-STAT3 antibody (rabbit polyclonal to p-STAT3 (Tyr705) (ab30646), Abcam) for one hour at room temperature, slides were thoroughly washed three times with PBS. Bound antibodies were detected using the EnVision Detection Systems Peroxidase/DAB, Rabbit/Mouse kit (Dako, Glostrup, Denmark). The slides were then counterstained with hematoxylin. Paraffin-embedded sections of human breast carcinoma tissues and normal lymph node tissues were used as positive controls for STAT3 and SHP-1, respectively, as described in the datasheet provided by the manufacturer. Negative controls had the primary antibody replaced by PBS. STAT3 and SHP-1 immunoreactivity was scored as negative, weak, moderate and strong expression, respectively.

This study was approved by the ethics committee of the Institutional Review Board of Taipei Veterans General Hospital. Informed consent was obtained from all sample donors at the time of their donation in accordance with the Declaration of Helsinki.

### Statistical analysis

Data are expressed as mean ± SD or SE. Statistical comparisons were based on nonparametric tests and statistical significance was defined at *P* <0.05. All statistical analyses were performed using SPSS for Windows software, version 12.0 (SPSS, Chicago, IL, USA).

## Results

### SC-1 and SC-43 have no effects on raf-1 kinase activity and show more potent anti-proliferative activity than sorafenib in breast cancer cells

**Figure 1:**
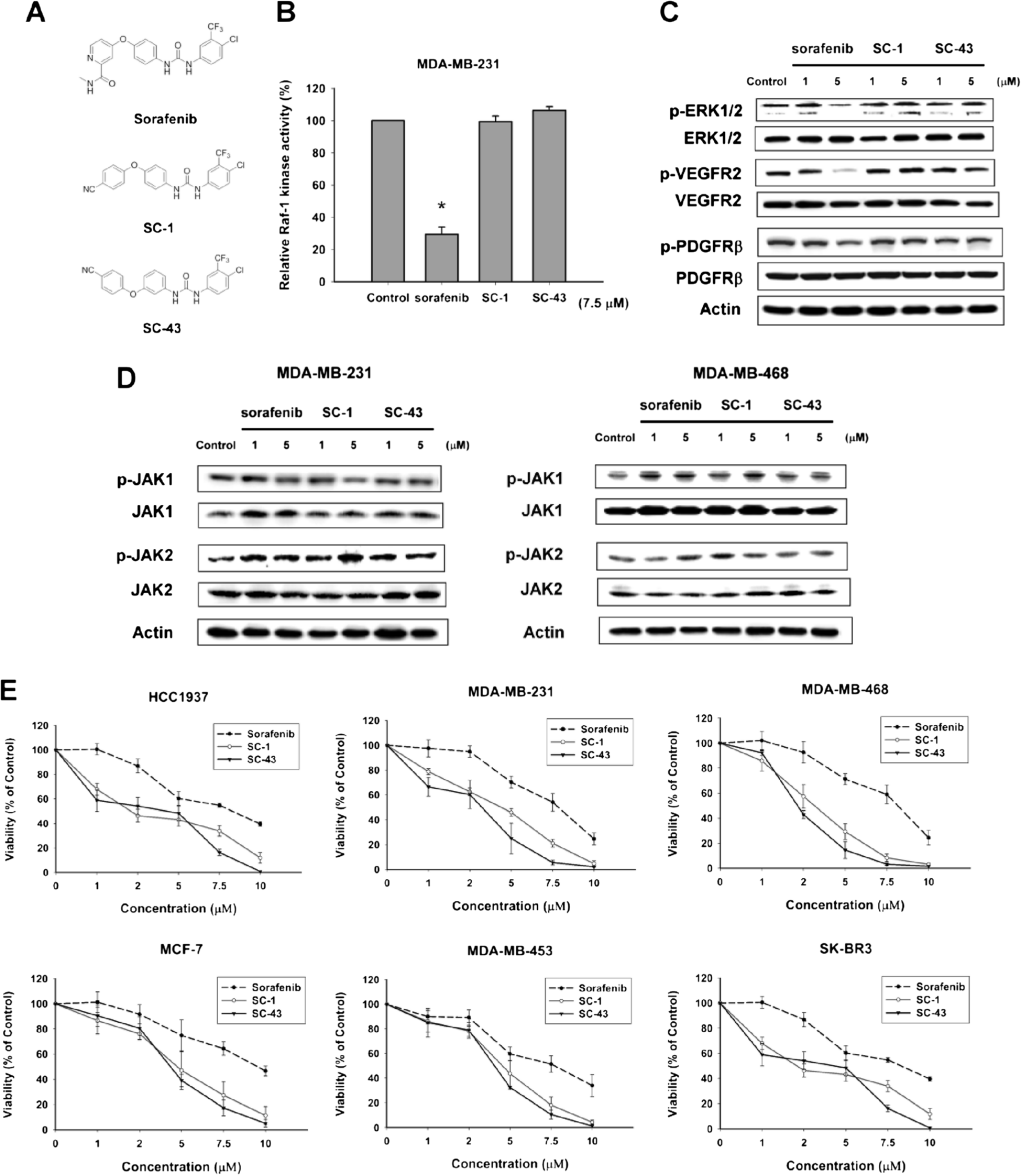
**SC-1 and SC-43, without effects on raf-1 kinase activity, show more potent anti-proliferative activity than sorafenib in breast cancer cells. A**, chemical structures of sorafenib, SC-1 and SC-43. **B**, effects of sorafenib, SC-1 and SC-43 on Raf-1 activity in MDA-MB-231 cells. Columns, mean (n = 3); bars, SD; *P <0.05 compared to control. **C***,* effects of sorafenib, SC-1 and SC-43 on the phosphorylation of ERK1/2, VEGFR2 and PDGFRβ in MDA-MB-231cells. Cells were exposed to sorafenib, SC-1 or SC-43 at 1 and 5 μM for 12 hours. Data are representative of three independent experiments. **D**, effects of sorafenib, SC-1 and SC-43 on the phosphorylation of STAT3 upstream kinases JAK1 and JAK2 in MDA-MB-231 (Left) and MDA-MB-468 cells (Right). Cells were exposed to sorafenib, SC-1 or SC-43 at 1 and 5 μM for 12 hours. Data are representative of three independent experiments. **E**, dose-escalation effects of sorafenib, SC-1 and SC-43 on cell viability in six breast cancer cell lines. Cells were exposed to sorafenib, SC-1 or SC-43 at the indicated doses for 48 hours and cell viability was assessed by the MTT assay. Points, mean (n = 3); bars, SD. MTT, 3-(4,5-dimethylthiazol-2-yl)-2,5-diphenyltetrazolium bromide.

Sorafenib analogues SC-1 and SC-43 are structurally similar to sorafenib, but are modified by replacement of the N-methylpicolinamide with a phenylcyano group (Figure 1A). In agreement with our previous reports [23,24], SC-1 and SC-43 demonstrated no effects on raf-1 kinase activity (Figure 1B). In MDA-MB-231 cells, sorafenib, but not SC-1 or SC-43, significantly suppressed p-ERK1/2 expression downstream of Raf-1, indicating raf-1 kinase inhibition by sorafenib, but not by SC-1 or SC-43 (Figure 1C). Moreover, SC-1 and SC-43 did not inhibit the phosphorylation of VEGFR2 and PDGFRβ, as did sorafenib (Figure 1C). Next, we examined the effects of SC-1 and SC-43 on STAT3 upstream kinases including JAK1 and JAK2. As shown in Figure 1D, sorafenib and its analogues SC-1 and SC-43 showed no significant effects on the phosphorylation of JAK1 or JAK2 in MDA-MB-231 and MDA-MB-468 cells (Figure 1D). To investigate the antitumor effects of SC-1 and SC-43 on breast cancer cells, we assessed the antiproliferative activity of sorafenib and its analogues in a panel of six human breast cancer cell lines: TNBC cells HCC-1937, MDA-MB-231 and MDA-MB-468; HER2-overexpressing cells MDA-MB-453 and SK-BR3; and estrogen receptor positive cells MCF-7 (Figure 1E). As shown in Figure 1E, both SC-1 and SC-43 demonstrated dose-dependent suppression of cell viability that was more effective than that of sorafenib in all tested breast cancer cells (Figure 1E).

### SC-1 and SC-43 demonstrate more potent apoptotic activity and p-STAT3 inhibition than sorafenib in breast cancer cells

**Figure 2:**
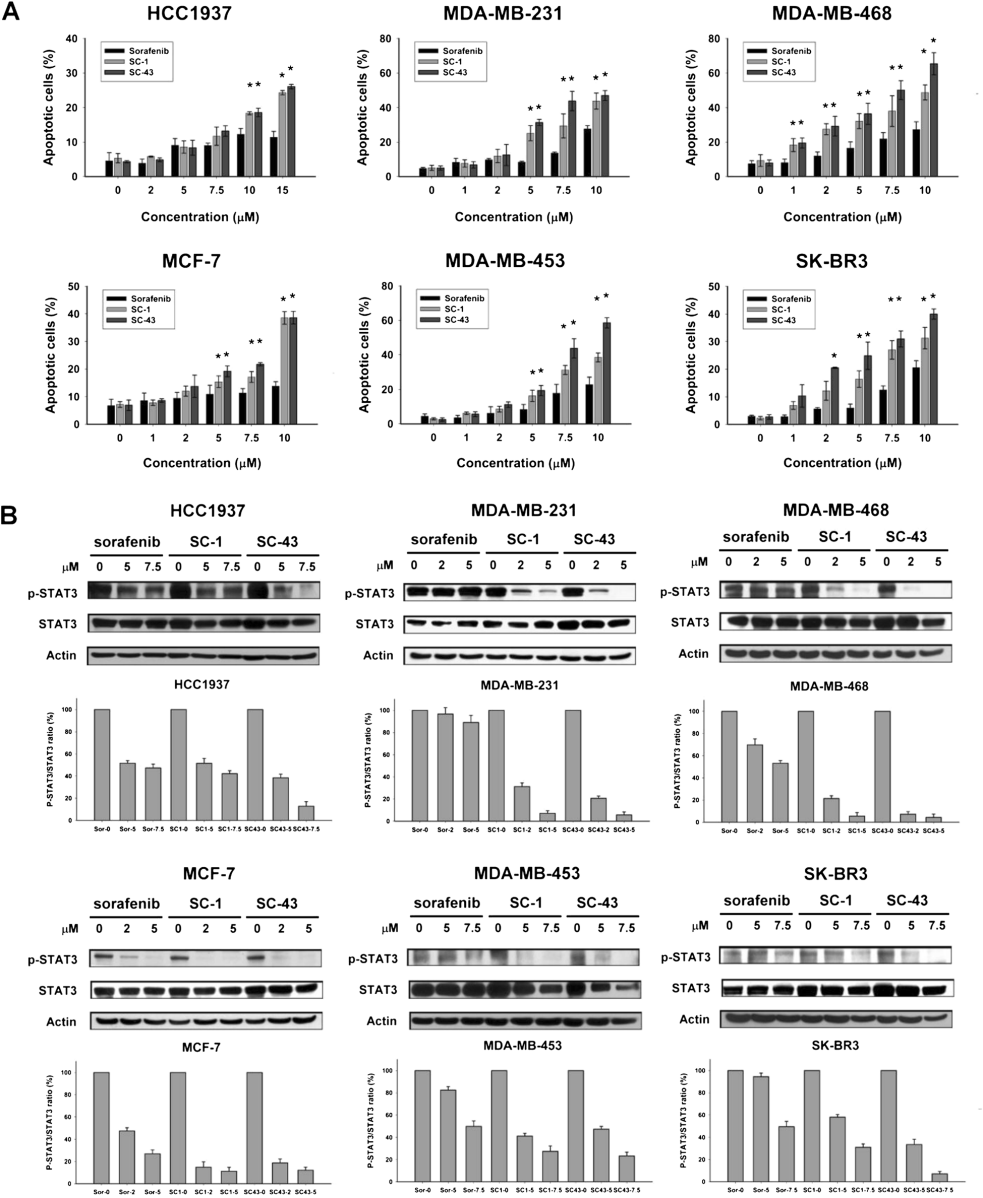
**SC-1 and SC-43 demonstrate more potent apoptosis and p-STAT3 inhibition than sorafenib in breast cancer cells. A**, dose-escalation effects of sorafenib, SC-1 and SC-43 on apoptosis in six breast cancer cell lines. Cells were exposed to sorafenib, SC-1 or SC-43 at the indicated doses for 36 hours. Apoptotic cells were analyzed by flow cytometry. Points, mean; bars, SD (n = 3). *P <0.05 compared to sorafenib treatment at each indicated dose. **B**, dose-dependent analysis of p-STAT3 and STAT3 in drug treated breast cancer cells. Cells were exposed to sorafenib, SC-1 or SC-43 at the indicated doses for 36 hours. Cell lysates were prepared and assayed for these molecules by western blotting. Western blot data are representative of three independent experiments. The ratio of p-STAT3 to STAT3 is shown below each western blot data set for each cell line. Immunoblots were scanned by a UVP BioSpectrum AC image system and quantitated using VisionWork LS software. Columns, mean (n = 3); bars, SD.

Flow cytometry analysis of sub-G1 cells showed that SC-1 and SC-43 consistently induced more potent apoptotic activity than sorafenib at the same indicated doses in the six breast cancer cell lines (Figure 2A). In addition, SC-1 and SC-43 showed differential apoptotic activity among different human breast cancer cell lines. In HCC1937 cells, higher doses of SC-1 and SC-43 were required to show significant cytotoxic efficacy, in comparison with the other cell lines. Our previous reports confirmed sorafenib as a p-STAT3 inhibitor [12,22-25]; here, we examined the effect of SC-1 and SC-43 on p-STAT3 at Tyr705 in comparison with sorafenib. As shown in Figure 2B, SC-1 and SC-43 showed greater inhibition of p-STAT3 than sorafenib at the same indicated doses in the tested breast cancer cells. In general, the extent of p-STAT3 inhibition corresponded with the extent of apoptosis induced by these agents within each cell line. It should be noted that there was some variation in drug dose-effect on p-STAT3 inhibition among different cell lines: SC-43 significantly suppressed p-STAT3 expression at 5 μM in MDA-MB-231, MDA-MB-468 and MCF-7, whereas it significantly suppressed p-STAT3 at 7.5 μM in the other three cell lines (Figure 2B).

### Downregulation of p-STAT3 contributes the apoptotic effects of SC-1 and SC-43 in breast cancer cells

**Figure 3:**
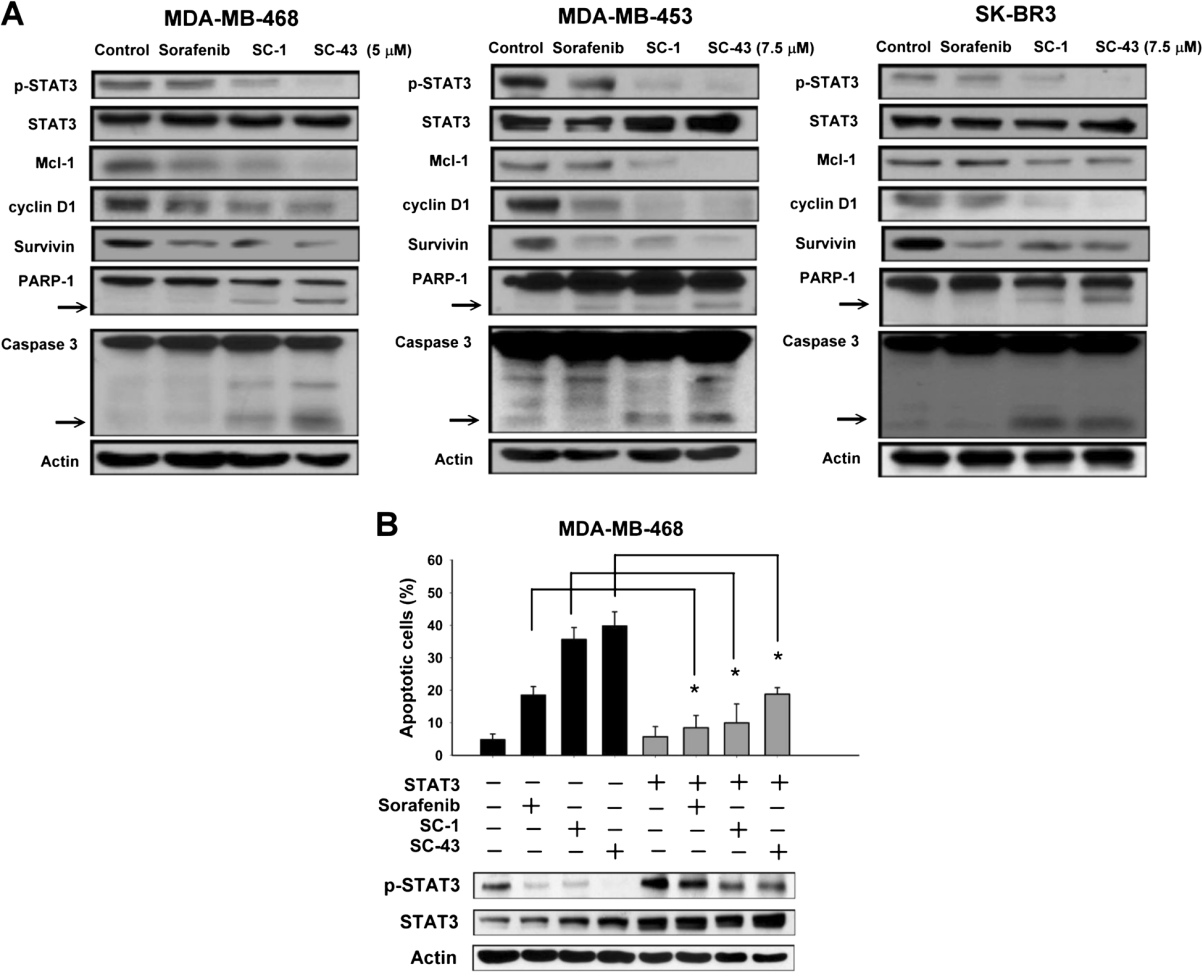
**Downregulation of p-STAT3 contributes apoptotic effects of SC-1 and SC-43 in breast cancer cells. A**, analysis of molecular effects of sorafenib, SC-1 and SC-43 on STAT3-related proteins, PARP-1 and caspase-3. Cells were exposed to sorafenib, SC-1 or SC-43 at doses of 5 μM for MDA-MB-468 cells, and 7.5 μM for MDA-MB-453 and SK-BR3 cells for 36 hours. Cell lysates were prepared and assayed for these molecules by western blotting. Western blot data are representative of three independent experiments. Apoptosis is evidenced by cleavage of PARP-1 or caspase-3. Arrows indicate cleavage forms of caspase-3 or PARP-1. **B**, protective effect of STAT3 on apoptosis induced by sorafenib, SC-1 or SC-43 in MDA-MB-468 cells. Cells (wild type or ectopic expression of STAT3) were exposed to sorafenib, SC-1 and SC-43 at 5 μM for 36 hours. Apoptotic cells were analyzed by flow cytometry. Columns, mean; bars, SD (n = 3). *P <0.05.

We examined the molecular signaling downstream of p-STAT3 in drug-treated breast cancer cells to confirm that p-STAT3 plays a major role in the apoptotic effects caused by SC-1 and SC-43 in breast cancer cells. As shown in Figure 3A, SC-1 and SC-43 downregulated p-STAT3 as well as the downstream effectors driven by STAT3, such as Mcl-1, cyclin D1 and survivin. Induction of apoptosis was evidenced by the activation of caspase-3 and PARP cleavage in drug-treated cells (Figure 3A). To further validate the role of STAT3 in SC-1- and SC-43-induced apoptosis in breast cancer cells, we next generated MDA-MB-468 cells with stable expression of STAT3. As shown in Figure 3B, ectopic expression of STAT3 reversed downregulation of p-STAT3 and reduced the apoptosis caused by sorafenib, SC-1 and SC-43 in MDA-MB-468 cells, suggesting that STAT3 mediates the apoptotic effects of these agents in breast cancer cells.

### SHP-1-dependent inhibition of STAT3 mediates apoptosis caused by SC-1 and SC-43 in breast cancer cells

**Figure 4:**
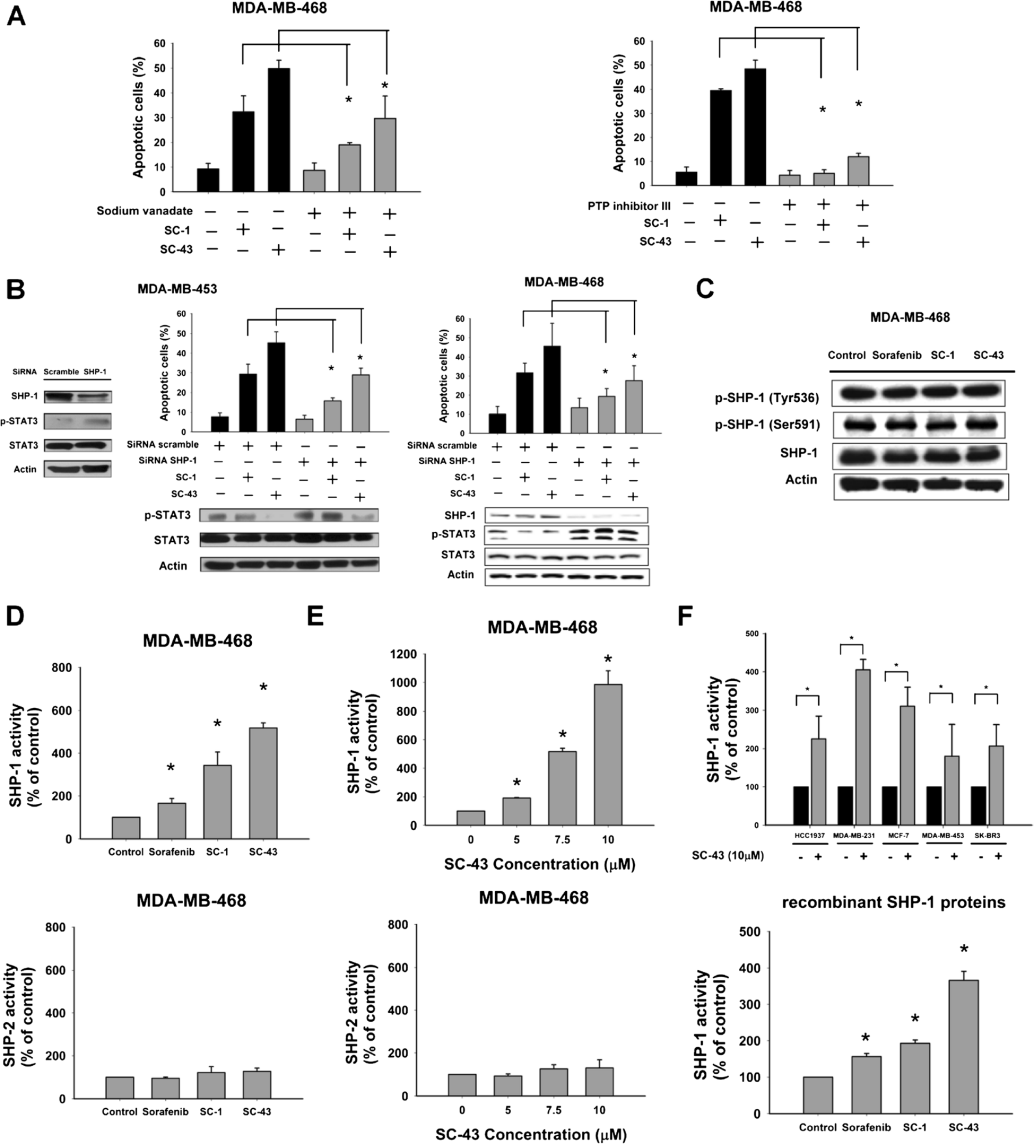
**SHP-1-dependent inhibition of STAT3 mediates the apoptotic effects of SC-1 and SC-43 in breast cancer cells. A**, inhibition of SHP-1 reverses effects of SC-1 and SC-43 on apoptosis. MDA-MB-468 cells were pretreated with 50 μM sodium vanadate, a non-specific phosphatase inhibitor (Left), or 50 μM PTP inhibitor III, a specific SHP-1 inhibitor (Right), for 60 minutes and then treated with SC-1 or SC-43 at 7.5 μM for 36 hours. **B**, silencing SHP-1 by siRNA reduces the effects of SC-1 and SC-43 on p-STAT3 inhibition and apoptosis in MDA-MB-453 and MDA-MB-468 cells. Cells were transfected with control siRNA (scrambled) or SHP-1 siRNA for 48 hours then treated with SC-1 or SC-43 at 7.5 μM for another 24 hours. **C**, effects of sorafenib, SC-1 and SC-43 on SHP-1 expression and phosphorylation (Tyr536 and Ser591). MDA-MB-468 cells were exposed to sorafenib, SC-1 or SC-43 at 7.5 μM for 36 hours. Western blot data are representative of three independent experiments. **D***,* the activity of SHP-1 (Upper) and SHP-2 (Lower) in drug-treated MDA-MB-468 cells. Cells were treated with sorafenib, SC-1 or SC-43 at 7.5 μM for 36 hours and cell lysates were assayed for phosphatase activity as described in Methods. **E**, dose-escalation effects of SC-43 on the activity of SHP-1 (Upper) and SHP-2 (Lower) in drug-treated MDA-MB-468 cells. Cells were treated with SC-43 at the indicated doses for 36 hours. **F**, effect of SC-43 on SHP-1 activity in drug-treated HCC-1937, MDA-MB-231, MCF-7, MDA-MB-453 and SK-BR3 cells (Upper); cells were treated with SC-43 at 10 μM for 36 hours. Effects of sorafenib, SC-1 and SC-43 on phosphatase activity in recombinant SHP-1 protein (Lower). Recombinant SHP-1 protein (25 ng) was incubated with drugs at 100 nM for 30 minutes. For bar charts, Columns, mean; bars, SD (*n* = 3). *P <0.05.

To further delineate the role of phosphatase in SC-1 and SC-43 induced apoptosis in breast cancer cells, first, we tested the effects of a general phosphatase inhibitor, sodium vanadate, and a SHP-1 phosphatase-specific inhibitor, PTP inhibitor III, on apoptosis-induction by SC-1 and SC-43 (Figure 4A). Our results showed that sodium vanadate repressed the percentage of apoptosis caused by SC-1 and SC-43 (Figure 4A, Left). Consistent with the result for sodium vanadate, the more specific SHP-1 inhibitor also rescued the effects of SC-1- and SC-43-induced cell death (Figure 4A, Right). Notably, the protective effect of the specific SHP-1 inhibitor was greater than that of sodium vanadate, implying that SHP-1 phosphatase is involved in SC-1- and SC-43-mediated cancer cell death. Moreover, both phosphatase inhibitors alone did not significantly induce apoptosis. Next, we knocked down SHP-1 by siRNA specific to SHP-1 in MDA-MB-453 and MDA-MB-468 cells (Figure 4B). As shown in Figure 4B, siRNA-mediated knockdown of SHP-1 reduced SC-1- and SC-43-mediated apoptosis and also restored the inhibition of p-STAT3 (Figure 4B). These data suggest an indispensable role for SHP-1 in the drug mechanism of SC-1 and SC-43. We next examined SHP-1 protein expression, SHP-1 phosphorylation and SHP-1 activity in drug treated MDA-MB-468 cells. We found that SC-1 and SC-43 did not significantly alter SHP-1 expression and SHP-1 phosphorylation (at Tyr536 and Ser591, sites known to affect SHP-1 function) [12] (Figure 4C); instead, SC-1 and SC-43 enhanced SHP-1 activity more than sorafenib in MDA-MB-468 cells (Figure 4D, upper). In contrast, SHP-2 activity was not significantly altered by sorafenib, SC-1 or SC-43 (Figure 4D, lower). Moreover, SC-43 demonstrated a dose-dependent escalation of SHP-1 activity in MDA-MB-468 cells, whereas SC-43 showed no significant effect on SHP-2 despite dose-escalation (Figure 4E). Similarly, we observed SC-43 enhanced SHP-1 activity in five other breast cancer cell lines (Figure 4F, upper). Since sorafenib, SC-1 and SC-43 enhanced SHP-1 activity without altering SHP-1 expression or phosphorylation, we further incubated these drugs with pure recombinant SHP-1 protein and assayed for SHP-1 activity. The results showed that sorafenib, SC-1 and SC-43 directly enhanced SHP-1 activity *in vitro* (Figure 4F, lower). Together these results show that SHP-1 participated in SC-1- and SC-43-induced downregulation of p-STAT3 and their subsequent apoptotic effects.

### Therapeutic evaluation of SC-1 and SC-43 on breast cancer xenograft tumor growth *in vivo*

**Figure 5:**
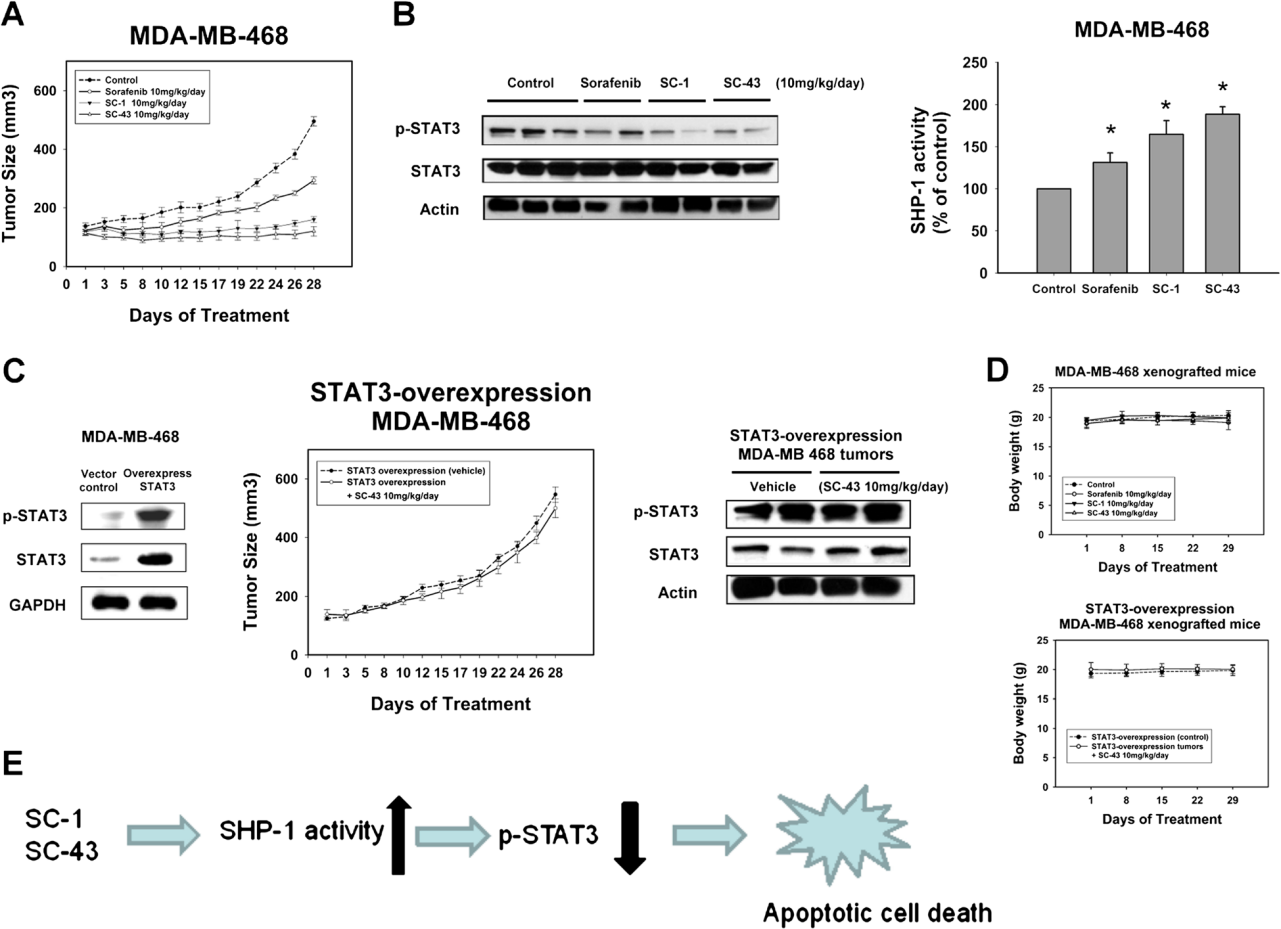
**Therapeutic evaluation of SC-1 and SC-43 on breast cancer xenograft tumor growth *in vivo*. A**, SC-1 and SC-43 show significant antitumor effects on MDA-MB-468 tumors. Growth curves of MDA-MB-468 tumors treated with vehicle, sorafenib, SC-1 and SC-43. Points, mean (n = 6); bars, SE. **B**, left, western blot analysis of p-STAT3 and STAT3 in MDA-MB-468 tumors. Right, the activity of SHP-1 in MDA-MB-468 tumors. Tumors were harvested 28 days after treatment and assayed for molecular events by western blotting and for SHP-1 activity. Columns, mean; bars, SD (n = 3). *P <0.05. **C**, overexpressed STAT3 in MDA-MB-468 tumors shows a significant protective effect against treatment with SC-43. Left*,* p-STAT3 and STAT3 expressions in STAT3-overexpressed and in empty vector- transfected MDA-MB-468 cells. Middle*,* growth curves of STAT3-overexpressed MDA-MB-468 tumors treated with vehicle and SC-43. Points, mean (n = 6); bars, SE. Right, western blot analysis of p-STAT3 and STAT3 in STAT3-overexpression MDA-MB-468 tumors treated with vehicle or SC-43. Tumors were harvested 28 days after treatment and assayed for molecular events by western blotting. **D**, body weight of xenograft mice bearing MDA-MB-468 tumors (left) and STAT3-overexpression MDA-MB-468 tumors (right) during the *in vivo* experiment. Points, mean (n = 6); bars, SE. Female NCr athymic nude mice (four to six weeks of age) were used for experiments (A) to (D). Mice were treated with sorafenib, SC-1 or SC-43 (10 mg/kg body weight) per os daily. Controls received vehicle. **E**, scheme of SHP-1 mediated drug mechanism of SC-1 and SC-43 in breast cancer cells.

To verify the therapeutic effects of SC-1 and SC-43 as potentially clinically useful, we evaluated the *in vivo* efficacy of sorafenib and its analogues in tumor-bearing mice. As shown in Figure 5A, sorafenib, SC-1 and SC-43 at the same dose of 10 mg/kg/day all significantly inhibited MDA-MB-468 xenograft tumor growth. Notably, both SC-43 and SC-1 exhibited tumor-growth inhibition that was superior to that of sorafenib (Figure 5A). Importantly, tumor-growth inhibition was correlated with p-STAT3 inhibition (Figure 5B, left), as well as with the enhancement of SHP-1 activity in these drug-treated xenograft tumors (Figure 5B, right). Next, we prepared MDA-MB-468 cells with ectopic expression of STAT3 (Figure 5C, left) and inoculated nude mice with the STAT3-overexpressed cancer cells. We further tested SC-43 efficacy in mice bearing STAT3-overexpressed MDA-MB-468 xenograft tumors (Figure 5C, middle). Treatment with SC-43 at 10 mg/kg/day did not significantly inhibit the tumor growth of STAT3-overexpressed MDA-MB-468 tumors and failed to suppress p-STAT3 expression in these tumors (Figure 5C, right), indicating the pivotal role of downregulation of p-STAT3 in mediating the drug effects of SC-43. In addition, there were no apparent differences in body weight or toxicity in the drug-treated mice in comparison with the control group (Figure 5D). Taken together, these results confirm that sorafenib analogues SC-1 and SC-43 increased SHP-1 activity which downregulated p-STAT3 and led to tumor inhibition in a breast cancer xenograft model. A schema of the drug mechanism of SC-1 and SC-43 is shown in Figure 5E.

### Expression of SHP-1 and p-STAT3 in breast tumor tissue from breast cancer patients

**Figure 6:**
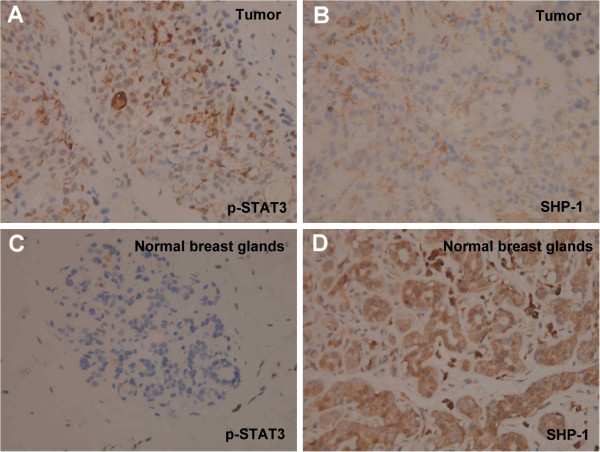
**Immunohistochemical stain for p-STAT3 and SHP-1 in a clinical sample.** Representative immunohistochemical patterns showing **(A)** strong nuclear expression with no cytoplasmic expression for STAT3 in cancer cells, **(B)** no nuclear expression with mild cytoplasmic expression for SHP-1 in cancer cells, **(C)** neither nuclear expression nor cytoplasmic expression for STAT3 in adjacent normal breast tissue, and **(D)** no nuclear expression and strong cytoplasmic expression for SHP-1 in adjacent normal breast tissue.

In breast cancer cells from representative breast tumor tissue from a breast cancer patient, p-STAT3 showed prominent nuclear expression and negative cytoplasmic expression (Figure 6, upper left), but negative nuclear and cytoplasmic expression compared to adjacent normal breast cells (Figure 6, lower left). On the contrary, SHP-1 showed weak cytoplasmic expression with negative nuclear expression in breast cancer cells (Figure 6, upper right), but strong cytoplasmic expression with negative nuclear expression in adjacent normal breast cells (Figure 6, lower right).

Further studies as well as more samples are warranted to clarify the relationship between SHP-1 and p-STAT3 expression in various subtypes of breast cancer.

## Discussion

This study reveals that two sorafenib analogues, SC-1 and SC-43, that do not inhibit raf-1 kinase activity, show better anti-cancer effects in human breast cancer cells than sorafenib and that this improved efficacy is mediated by SHP-1-dependent p-STAT3 inhibition. Structurally, both SC-1 and SC-43 are very similar (Figure 1A) and lack hydrogen donor ability as the pyridine ring and amide functional group on sorafenib has been replaced with phenyl cyanide. The pyridine ring and amide functional group is a key structure of sorafenib that forms a hydrogen bond with b-Raf kinase in the ATP binding pocket [26]. Here, we found that these two analogues with this functional group removed cause a cell death effect that surpasses that of sorafenib (Figure 1 and Figure 2). Our data not only confirmed that p-STAT3 is a target of sorafenib, but also suggested that the extent of p-STAT3 inhibition may be correlated with drug potency in these p-STAT3 inhibitors (Figure 3). Furthermore, we demonstrated that p-STAT3 inhibition by SC-1 and SC-43 is attributed to increased SHP-1 activity by these agents (Figure 4). Importantly, this SHP-1 dependent drug mechanism of SC-1 and SC-43 was validated in a breast cancer xenograft tumor model (Figure 5).

SHP-1 is a non-receptor phosphatase that negatively regulates cytokine signaling, such as that of IL-3R, the PDGF- and EGF receptors and others [27-29]. SHP-1 expression is diminished or abolished in most leukemia and lymphoma cell lines and tissues and in some non-hematopoietic cancer cell lines, such as estrogen-receptor negative breast cancer cell lines and some colorectal cancer cell lines [30-32]. Moreover, SHP-1 has been shown to be necessary for receptor-mediated cytotoxic signaling and ectopically expressed SHP-1 has been shown to reduce cell proliferation in breast cancer cells [31,33]. Conversely, siRNA knockdown of SHP-1 expression in prostate cancer cells resulted in increased cellular proliferation [34]. In light of the tumor suppressive function of SHP-1, enhancing its activity may be a promising strategy for cancer therapy [32]. As mentioned earlier, there are an increasing number of reports of agents that can act as ‘SHP-1 enhancers’ to kill cancer cells [14-21]. Interestingly, we recently discovered that dovitinib (formerly TKI258), a novel multi-kinase inhibitor that targets VEGFR1-3, PDGFR-β and FGFR1-3, as well as FLT-3, c-KIT, Ret, TrkA and csf-1 [35], could also induce apoptosis and overcome sorafenib resistance through SHP-1-mediated p-STAT3 inhibition in hepatocellular carcinoma cells [36,37]. Our results further strengthen the evidence that targeting p-STAT3 by enhanced SHP-1 activity may have anti-cancer potential.

Our study also highlights the feasibility of targeting SHP-1 dependent p-STAT3 inhibition in breast cancer therapy. Although currently there are several well-known targeted agents for HER2-overexpressing breast cancer subtype and hormone antagonists for hormone receptor-positive breast cancer subtypes, the TNBC subtype is still in need of targeted agents. In addition, drug resistance to current therapy remains a significant problem at late stage and, therefore, new therapy is always needed. Furthermore, as an oral multi-kinase inhibitor with anti-angiogenic and anti-proliferative activity, sorafenib only demonstrated modest efficacy in Phase II trials which indicates a potential role for sorafenib in combination with select chemotherapies for HER2-negative advanced breast cancers [38]. Data from clinical trials have shown that the anti-angiogenesis strategy has limited survival benefit in metastatic breast cancer, and anti-angiogenesis agents have generally been developed for use in combination with chemotherapies [39,40]. Our data further provide an alternative explanation of why sorafenib exerts only limited clinical anti-breast cancer activity. Here, we showed that sorafenib did not efficiently increase SHP-1 activity and did not effectively inhibit p-STAT3 in several breast cancer cell lines such as MDA-MB-231 and MDA-MB-468 cells (Figure 2) or in MDA-MB-468 xenograft tumors (Figure 5A), as compared with sorafenib analogues SC-1 and SC-43. In contrast, sorafenib analogues SC-1 and SC-43, with enhanced p-STAT3 inhibition in comparison with sorafenib, that is, enhanced SHP-1 activity, resulted in more potent apoptotic activity and afforded better protection against xenograft tumor growth than sorafenib. Further studies are necessary to validate the therapeutic relevance of these novel SHP-1-activating agents in breast cancer therapy.

The role of SHP-1 in clinical breast tumor tissue is another interesting subject that may be potentially therapeutically relevant. Previously, Yip *et al.*[30] reported that SHP-1 mRNA seemed inversely correlated with estrogen receptor positivity in breast cancer cell lines and that up to 58% (42/72) of primary breast cancer tissues showed increased SHP-1 mRNA expression. Recently, Insabato *et al.*[41] analyzed SHP-1 expression by immunohistochemistry in a breast tissue microarray composed of 2,081 cores (68% of which were invasive ductal carcinoma) and found an approximate 7.2% SHP-1 positive rate for all breast tumor tissue. They also found that SHP-1 expression correlated directly with expression of HER2 (11% and 18% SHP-1 positive rates in HER2 immunohistochemical staining 2+ and 3+ samples, respectively) and inversely with expression of the estrogen receptor (13% versus 5% in estrogen receptor-negative and positive samples, respectively) and concluded that SHP-1 expression was confined to a well-defined subset of high-grade breast tumors [41]. Ambiguously, while the endogenous SHP-1 expression level might be implicated as a prognostic indicator [41], whether endogenous SHP-1 expression is a biomarker of drug efficacy remains to be clarified. Recently SHP-1 has been shown to play a prominent role as a determinant of imatinib treatment resistance in chronic myeloid leukemia cell lines; SHP-1 expression is significantly lower in resistant than in sensitive cell lines and ectopic expression of SHP-1 restores drug sensitivity [42]. It is possible that the ability to enhance SHP-1 activity, rather than baseline SHP-1 expression, will reflect drug efficacy of agents that target SHP-1-mediated p-STAT3 inhibition. Although we have shown here that a representative breast tumor tissue has reciprocal expression of SHP-1 and p-STAT3 in cancer cells and adjacent non-cancer breast tissue (Figure 6), large immunohistochemistry-based studies are needed to address the role of SHP-1 expression in relation to p-STAT3 in such a heterogeneous disease comprehensively.

The current results notwithstanding, the detailed mechanisms by which sorafenib and its analogues enhance SHP-1 activity remain to be elucidated. SHP-1 is composed of a catalytic domain at the C-terminus and two SH2 domains at the N-terminus for phosphotyrosine binding. It has been shown that an autoinhibitory conformation occurs between the SH2 domain at the N-terminal and the catalytic PTP domain [43-45] and that the catalytic PTP loop for autoinhibition is critical for SHP-1 phosphatase activity. Our data show that SC-1 and SC-43 may directly increase SHP-1 activity without altering SHP-1 expression or phosphorylation (Figure 4). We validate that SHP-1-dependent p-STAT3 inhibition clearly plays a role in SC-1- and SC-43-induced apoptosis. Moreover, there are several known substrates of SHP-1 in different cell types, notably in hematopoietic cells [32,46-48]. For example, JAK2 kinase and STAT5 in erythropoietic cells [46], c-KIT kinase in hematopoietic cells [47] and nerve growth factor receptor TrkA in neuron cells [48]. However, there is limited data showing SHP-1 substrates other than p-STAT3 in breast cancer cells. Further mechanistic studies are definitely needed for determining the effects of SC-1 and SC-43 on other potential SHP-1 substrates in breast cancer cells. In addition, although SC-1 and SC-43 did not significantly alter the phosphorylation of STAT3 upstream kinases JAK1 and JAK2 (Figure 1D), the role of other kinases in the inactivation of STAT3 cannot be completely excluded based on current available data and further studies are necessary.

## Conclusions

In summary, our results suggest that novel sorafenib analogues SC-1 and SC-43 induce apoptosis through SHP-1 dependent STAT3 inactivation and demonstrate more potent apoptotic activities than sorafenib in human breast cancer cells. Targeting p-STAT3 by enhancement of SHP-1 activity may be a novel therapeutic approach for breast cancer.

## Abbreviations

(D)MEM: (Dulbecco’s) modified Eagle’s medium; ALK: Anaplastic lymphoma kinase; DMSO: Dimethyl sulfoxide; EGF: Epidermal growth factor; ERK1/2: Extracellular signal-regulated protein kinases 1 and 2; FGFR: Fibroblast growth factor receptor; FLT-3: FMS-like tyrosine kinase 3; HER2: Human epidermal growth factor receptor 2; IL: Interleukin; IL-3R: Interleukin-3 receptor; JAK: Janus kinase; MEK: Mitogen-activated protein kinase kinase; MTT: 3-(4,5-Dimethylthiazol-2-yl)-2,5-diphenyltetrazolium bromide; PARP: Poly ADP-ribose polymerase; PBS: Phosphate-buffered saline; PDGFR: Platelet derived growth factor receptor; PTP: Protein tyrosine phosphatase; SHP: Src homology 2-domain containing tyrosine phosphatase; siRNA: Small interfering RNA; STAT3: Signal transducers and activators of transcription 3; TNBC: Triple-negative breast cancer; TRAIL: Tumor necrosis factor-related apoptosis-inducing ligand; VEGFR: Vascular endothelial growth factor receptor.

## 

© 2013 Liu et al.; licensee BioMed Central Ltd. This is an open access article distributed under the terms of the Creative Commons Attribution License (http://creativecommons.org/licenses/by/2.0) which permits unrestricted use, distribution, and reproduction in any medium, provided the original work is properly cited.

## Competing interests

The authors declare that they have no competing interests.

## Authors’ contributions

CWS and KFC were responsible for coordination and manuscript editing as well as acting as corresponding authors. CYL, LMT, KFC and CWS participated in research design. LMT, KCC, CYL, PYC, JCS and WTT conducted experiments. LMT, CYL, KFC and CWS performed data analysis. LMT, CYL and KCC wrote or contributed to the writing of the manuscript. All authors have read and approved the final manuscript
