# Erratum to: Novel sorafenib analogues induce apoptosis through SHP-1 dependent STAT3 inactivation in human breast cancer cells

**DOI:** 10.1186/s13058-017-0800-2

**Published:** 2017-01-11

**Authors:** Chun-Yu Liu, Ling-Ming Tseng, Jung-Chen Su, Kung-Chi Chang, Pei-Yi Chu, Wei-Tien Tai, Chung-Wai Shiau, Kuen-Feng Chen

**Affiliations:** 1 Institute of Biopharmaceutical Sciences, National Yang-Ming University, No. 155 Sec. 2, Li-Nong Street, Taipei, 112 Taiwan; 2 School of Medicine, National Yang-Ming University, No. 155, Sec. 2, Li-Nong Street, Taipei, 112 Taiwan; 3 Division of Hematology and Oncology, Taipei Veterans General Hospital, No 201, Sec. 2, Shih-Pai Road, Taipei, 112 Taiwan; 4 Department of Medicine, Taipei Veterans General Hospital, No. 201, Sec. 2, Shih-Pai Road, Taipei, 112 Taiwan; 5 Department of Surgery, Taipei Veterans General Hospital, No. 201, Sec. 2, Shih-Pai Road, Taipei, 112 Taiwan; 6 Department of Pathology, St. Martin De Porres Hospital, No. 565, Sec. 2, Daya Road, Chiayi, 600 Taiwan; 7 Department of Medical Research, National Taiwan University Hospital, No. 7, Chung-Shan South Road, Taipei, 100 Taiwan; 8 National Center of Excellence for Clinical Trial and Research, National Taiwan University Hospital, No. 7, Chung-Shan South Road, Taipei, 100 Taiwan

## Erratum

In the published article [1], the authors noticed an error to Fig. 1e in which the MTT curve of drug treatments (sorafenib, SC-1 and SC-43) in SK-BR3 cells was erroneously put as the same with that of HCC-1937 cells.

The correct version of Fig. 1 (including correct Fig. 1e) is included in this erratum.

**Fig. 1:**
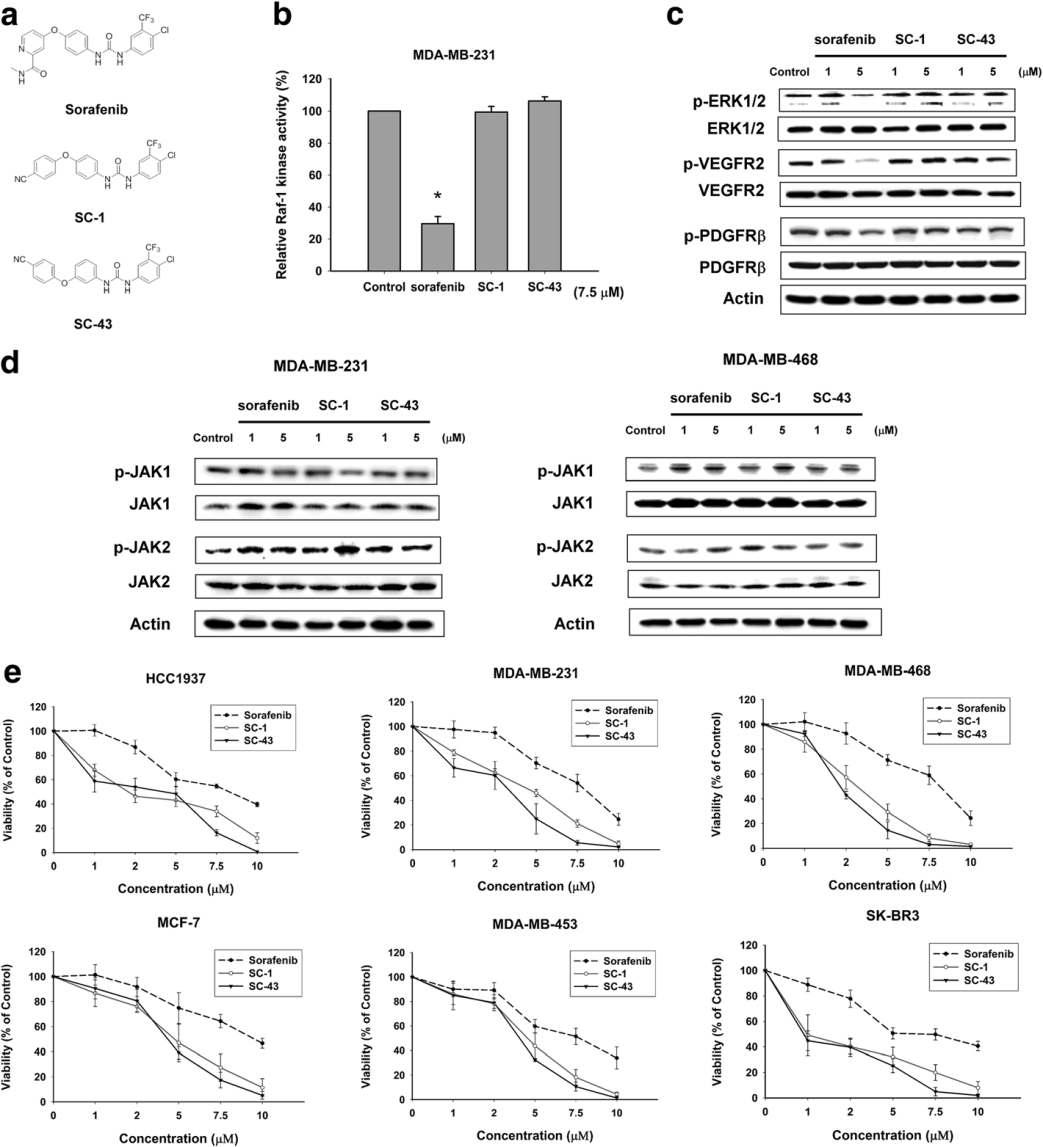
SC-1 and SC-43, without effects on raf-1 kinase activity, show more potent anti-proliferative activity than sorafenib in breast cancer cells. **a** chemical structures of sorafenib, SC-1 and SC-43. **b** effects of sorafenib, SC-1 and SC-43 on Raf-1 activity in MDA-MB-231 cells. Columns, mean (n = 3); bars, SD; *P <0.05 compared to control. **c** effects of sorafenib, SC-1 and SC-43 on the phosphorylation of ERK1/2, VEGFR2 and PDGFRβ in MDA-MB-231cells. Cells were exposed to sorafenib, SC-1 or SC-43 at 1 and 5 μM for 12 hours. Data are representative of three independent experiments. **d** effects of sorafenib, SC-1 and SC-43 on the phosphorylation of STAT3 upstream kinases JAK1 and JAK2 in MDA-MB-231 (Left) and MDA-MB-468 cells (Right). Cells were exposed to sorafenib, SC-1 or SC-43 at 1 and 5 μM for 12 hours. Data are representative of three independent experiments. **e** dose-escalation effects of sorafenib, SC-1 and SC-43 on cell viability in six breast cancer cell lines. Cells were exposed to sorafenib, SC-1 or SC-43 at the indicated doses for 48 hours and cell viability was assessed by the MTT assay. Points, mean (n = 3); bars, SD. MTT, 3-(4,5-dimethylthiazol-2-yl)-2,5-diphenyltetrazolium bromide
